# Supplementary material for: A Protein-Based Material from a New Approach Using Whole Defatted Larvae, and Its Interaction with Moisture
Source: Polymers (Basel). 2019 Feb 8;11(2):287. doi: 10.3390/polym11020287 (PMC6419081; doi:10.3390/polym11020287)
Supplement: Supplementary file 1 [file polymers-11-00287-s001.pdf]

Rapport utfärdad av
ackrediterat laboratorium

Report issued by
Accredited Laboratory

SWEDAC ACKREDITERING 1977 ISO/IEC 17025

Eurofins Food & Agro Testing Sweden AB
Box 887
Sjöhagsg. 3
SE-53119 Lidköping
www.eurofins.se

Tlf: +46 10 490 8310

Sveriges Lantbruksuniversitet
Cecilia Lalander
Lennart Hjelms väg 9
750 07 UPPSALA

AR-14-LW-016807-01

EUSELI-00064229

Kundnummer: LW8412219

Analysrapport

Provnummer:	525-2014-04160278
Provmärkning:	A02.M.1
Provet ankom:	2014-04-16
Analysrapport klar:	2014-04-30
Analyserna påbörjades:	2014-04-17 16:38:33

	Analys	Resultat	Enhet	Mäto.	Metod/ref	Lab
LP022	Råprotein enl. Dumas (Nx6.25)	**44.3**	g/100 g	± 7%	Dumas	*EUSELI*
LP00W	Alanin	**25.2**	g/kg	± 8%	SS-EN ISO 13903:2005	*EUSELI*
LP00W	Arginin	**21.8**	g/kg	± 8%	SS-EN ISO 13903:2005	*EUSELI*
LP00W	Asparaginsyra (asparagin och asparaginsyra)	**41.0**	g/kg	± 8%	SS-EN ISO 13903:2005	*EUSELI*
LP00W	Cystin	**2.6**	g/kg	± 8%	SS-EN ISO 13903:2005	*EUSELI*
LP00W	Fenylalanin	**15.5**	g/kg	± 8%	SS-EN ISO 13903:2005	*EUSELI*
LP00W	Glutaminsyra	**44.2**	g/kg	± 8%	SS-EN ISO 13903:2005	*EUSELI*
LP00W	Glycin	**25.4**	g/kg	± 8%	SS-EN ISO 13903:2005	*EUSELI*
LP00W	Histidin	**15.6**	g/kg	± 8%	SS-EN ISO 13903:2005	*EUSELI*
LP00W	Hydroxiprolin	**<0.1**	g/kg	± 8%	SS-EN ISO 13903:2005	*EUSELI*
LP00W	Isoleucin	**20.4**	g/kg	± 8%	SS-EN ISO 13903:2005	*EUSELI*
LP00W	Leucin	**30.1**	g/kg	± 8%	SS-EN ISO 13903:2005	*EUSELI*
LP00W	Lysin	**28.4**	g/kg	± 8%	SS-EN ISO 13903:2005	*EUSELI*
LP00W	Metionin	**7.8**	g/kg	± 8%	SS-EN ISO 13903:2005	*EUSELI*
LP00W	Ornitin	**0.1**	g/kg	± 8%	SS-EN ISO 13903:2005	*EUSELI*
LP00W	Prolin	**22.3**	g/kg	± 8%	SS-EN ISO 13903:2005	*EUSELI*
LP00W	Serin	**18.2**	g/kg	± 8%	SS-EN ISO 13903:2005	*EUSELI*
LP00W	Treonin	**16.5**	g/kg	± 8%	SS-EN ISO 13903:2005	*EUSELI*
LP00W	Tyrosin	**37.6**	g/kg	± 8%	SS-EN ISO 13903:2005	*EUSELI*
LP00W	Valin	**27.8**	g/kg	± 8%	SS-EN ISO 13903:2005	*EUSELI*
LP00W	Summa aminosyror	**400.5**	g/kg		SS-EN ISO 13903:2005	*EUSELI*
LP00B	Tryptofan	**7.23**	g/kg	± 10%	SS-EN ISO 13904:2005	*EUSELI*

Laboratoriet/laboratorierna är ackrediterade av respektive lands ackrediteringsorgan. Ej ackrediterade analyser är markerade med *

Helena Larsson, Rapportansvarig

Denna rapport är elektroniskt signerad.

Utförande Laboratorium

EUSELI Eurofins Food & Agro Testing Sweden AB, Lidköping

Laboratoriet/laboratorierna är ackrediterade av respektive lands ackrediteringsorgan. Ej ackrediterade analyser är markerade med *

eurofins

Rapport utfärdad av
ackrediterat laboratorium

Report issued by
Accredited Laboratory

SWEDAC ACKREDITERING 1977 ISO/IEC 17025

Eurofins Food & Agro Testing Sweden AB
Box 887
Sjöhagsg. 3
SE-53119 Lidköping
www.eurofins.se

Tlf: +46 10 490 8310

Sveriges Lantbruksuniversitet
Cecilia Lalander
Lennart Hjelms väg 9
750 07 UPPSALA

AR-14-LW-016808-01

EUSELI-00064229

Kundnummer: LW8412219

Analysrapport

Provnummer:	525-2014-04160279
Provmärkning:	A02.M.2
Provet ankom:	2014-04-16
Analysrapport klar:	2014-04-30
Analyserna påbörjades:	2014-04-17 16:38:33

	Analys	Resultat	Enhet	Mäto.	Metod/ref	Lab
LP022	Råprotein enl. Dumas (Nx6.25)	**44.0**	g/100 g	± 7%	Dumas	*EUSELI*
LP00W	Alanin	**25.7**	g/kg	± 8%	SS-EN ISO 13903:2005	*EUSELI*
LP00W	Arginin	**22.2**	g/kg	± 8%	SS-EN ISO 13903:2005	*EUSELI*
LP00W	Asparaginsyra (asparagin och asparaginsyra)	**41.4**	g/kg	± 8%	SS-EN ISO 13903:2005	*EUSELI*
LP00W	Cystin	**2.6**	g/kg	± 8%	SS-EN ISO 13903:2005	*EUSELI*
LP00W	Fenylalanin	**16.6**	g/kg	± 8%	SS-EN ISO 13903:2005	*EUSELI*
LP00W	Glutaminsyra	**44.1**	g/kg	± 8%	SS-EN ISO 13903:2005	*EUSELI*
LP00W	Glycin	**25.8**	g/kg	± 8%	SS-EN ISO 13903:2005	*EUSELI*
LP00W	Histidin	**15.8**	g/kg	± 8%	SS-EN ISO 13903:2005	*EUSELI*
LP00W	Hydroxiprolin	**<0.1**	g/kg	± 8%	SS-EN ISO 13903:2005	*EUSELI*
LP00W	Isoleucin	**21.2**	g/kg	± 8%	SS-EN ISO 13903:2005	*EUSELI*
LP00W	Leucin	**30.4**	g/kg	± 8%	SS-EN ISO 13903:2005	*EUSELI*
LP00W	Lysin	**27.9**	g/kg	± 8%	SS-EN ISO 13903:2005	*EUSELI*
LP00W	Metionin	**7.9**	g/kg	± 8%	SS-EN ISO 13903:2005	*EUSELI*
LP00W	Ornitin	**0.2**	g/kg	± 8%	SS-EN ISO 13903:2005	*EUSELI*
LP00W	Prolin	**22.0**	g/kg	± 8%	SS-EN ISO 13903:2005	*EUSELI*
LP00W	Serin	**17.1**	g/kg	± 8%	SS-EN ISO 13903:2005	*EUSELI*
LP00W	Treonin	**16.1**	g/kg	± 8%	SS-EN ISO 13903:2005	*EUSELI*
LP00W	Tyrosin	**37.2**	g/kg	± 8%	SS-EN ISO 13903:2005	*EUSELI*
LP00W	Valin	**28.7**	g/kg	± 8%	SS-EN ISO 13903:2005	*EUSELI*
LP00W	Summa aminosyror	**403.0**	g/kg		SS-EN ISO 13903:2005	*EUSELI*
LP00B	Tryptofan	**7.39**	g/kg	± 10%	SS-EN ISO 13904:2005	*EUSELI*

Laboratoriet/laboratorierna är ackrediterade av respektive lands ackrediteringsorgan. Ej ackrediterade analyser är markerade med *

Förklaringar
* Ej ackrediterad analys

AR-003 v78
1.67 130516

Mäto: Mätosäkerhet

Mätosäkerheten, om inget annat anges, redovisas som utvidgad mätosäkerhet med täckningsfaktor 2. Undantag relaterat till analyser utförda utanför Sverige kan förekomma. Ytterligare upplysningar kan lämnas på begäran. Upplysning om mätosäkerhet och detektionsnivåer för mikrobiologiska analyser lämnas på begäran.

Denna rapport får endast återges i sin helhet, om inte utförande laboratorium i förväg skriftligen godkänt annat. Resultaten relaterar endast till det insända provet.

Helena Larsson, Rapportansvarig

Denna rapport är elektroniskt signerad.

Utförande Laboratorium

EUSELI	Eurofins Food & Agro Testing Sweden AB, Lidköping

Laboratoriet/laboratorierna är ackrediterade av respektive lands ackrediteringsorgan. Ej ackrediterade analyser är markerade med *

Förklaringar
* Ej ackrediterad analys

Mäto: Mätosäkerhet

Mätosäkerheten, om inget annat anges, redovisas som utvidgad mätosäkerhet med täckningsfaktor 2. Undantag relaterat till analyser utförda utanför Sverige kan förekomma. Ytterligare upplysningar kan lämnas på begäran. Upplysning om mätosäkerhet och detektionsnivåer för mikrobiologiska analyser lämnas på begäran.

Denna rapport får endast återges i sin helhet, om inte utförande laboratorium i förväg skriftligen godkänt annat. Resultaten relaterar endast till det insända provet.

eurofins

Rapport utfärdad av
ackrediterat laboratorium

Report issued by
Accredited Laboratory

SWEDAC ACKREDITERING
1977
ISO/IEC 17025

Eurofins Food & Agro Testing Sweden AB
Box 887
Sjöhagsg. 3
SE-53119 Lidköping
www.eurofins.se

Tlf: +46 10 490 8310

Sveriges Lantbruksuniversitet
Cecilia Lalander
Lennart Hjelms väg 9
750 07 UPPSALA

AR-14-LW-016809-01

EUSELI-00064229

Kundnummer: LW8412219

Analysrapport

Provnummer:	525-2014-04160280
Provmärkning:	A02.M.3
Provet ankom:	2014-04-16
Analysrapport klar:	2014-04-30
Analyserna påbörjades:	2014-04-17 16:38:33

	Analys	Resultat	Enhet	Mäto.	Metod/ref	Lab
LP022	Råprotein enl. Dumas (Nx6.25)	**44.4**	g/100 g	± 7%	Dumas	*EUSELI*
LP00W	Alanin	**25.0**	g/kg	± 8%	SS-EN ISO 13903:2005	*EUSELI*
LP00W	Arginin	**21.8**	g/kg	± 8%	SS-EN ISO 13903:2005	*EUSELI*
LP00W	Asparaginsyra (asparagin och asparaginsyra)	**40.7**	g/kg	± 8%	SS-EN ISO 13903:2005	*EUSELI*
LP00W	Cystin	**2.7**	g/kg	± 8%	SS-EN ISO 13903:2005	*EUSELI*
LP00W	Fenylalanin	**15.5**	g/kg	± 8%	SS-EN ISO 13903:2005	*EUSELI*
LP00W	Glutaminsyra	**44.4**	g/kg	± 8%	SS-EN ISO 13903:2005	*EUSELI*
LP00W	Glycin	**25.7**	g/kg	± 8%	SS-EN ISO 13903:2005	*EUSELI*
LP00W	Histidin	**15.6**	g/kg	± 8%	SS-EN ISO 13903:2005	*EUSELI*
LP00W	Hydroxiprolin	**<0.1**	g/kg	± 8%	SS-EN ISO 13903:2005	*EUSELI*
LP00W	Isoleucin	**20.3**	g/kg	± 8%	SS-EN ISO 13903:2005	*EUSELI*
LP00W	Leucin	**30.2**	g/kg	± 8%	SS-EN ISO 13903:2005	*EUSELI*
LP00W	Lysin	**28.6**	g/kg	± 8%	SS-EN ISO 13903:2005	*EUSELI*
LP00W	Metionin	**7.8**	g/kg	± 8%	SS-EN ISO 13903:2005	*EUSELI*
LP00W	Ornitin	**0.1**	g/kg	± 8%	SS-EN ISO 13903:2005	*EUSELI*
LP00W	Prolin	**22.9**	g/kg	± 8%	SS-EN ISO 13903:2005	*EUSELI*
LP00W	Serin	**18.4**	g/kg	± 8%	SS-EN ISO 13903:2005	*EUSELI*
LP00W	Treonin	**16.5**	g/kg	± 8%	SS-EN ISO 13903:2005	*EUSELI*
LP00W	Tyrosin	**39.6**	g/kg	± 8%	SS-EN ISO 13903:2005	*EUSELI*
LP00W	Valin	**27.4**	g/kg	± 8%	SS-EN ISO 13903:2005	*EUSELI*
LP00W	Summa aminosyror	**403.2**	g/kg		SS-EN ISO 13903:2005	*EUSELI*
LP00B	Tryptofan	**7.50**	g/kg	± 10%	SS-EN ISO 13904:2005	*EUSELI*

Laboratoriet/laboratorierna är ackrediterade av respektive lands ackrediteringsorgan. Ej ackrediterade analyser är markerade med *

Förklaringar
* Ej ackrediterad analys

Mäto: Mätosäkerhet

Mätosäkerheten, om inget annat anges, redovisas som utvidgad mätosäkerhet med täckningsfaktor 2. Undantag relaterat till analyser utförda utanför Sverige kan förekomma. Ytterligare upplysningar kan lämnas på begäran. Upplysning om mätosäkerhet och detektionsnivåer för mikrobiologiska analyser lämnas på begäran.

Denna rapport får endast återges i sin helhet, om inte utförande laboratorium i förväg skriftligen godkänt annat. Resultaten relaterar endast till det insända provet.

Helena Larsson, Rapportansvarig

Denna rapport är elektroniskt signerad.

Utförande Laboratorium

EUSELI Eurofins Food & Agro Testing Sweden AB, Lidköping

Laboratoriet/laboratorierna är ackrediterade av respektive lands ackrediteringsorgan. Ej ackrediterade analyser är markerade med *

Förklaringar
* Ej ackrediterad analys

Mäto: Mätosäkerhet

Mätosäkerheten, om inget annat anges, redovisas som utvidgad mätosäkerhet med täckningsfaktor 2. Undantag relaterat till analyser utförda utanför Sverige kan förekomma. Ytterligare upplysningar kan lämnas på begäran. Upplysning om mätosäkerhet och detektionsnivåer för mikrobiologiska analyser lämnas på begäran.

Denna rapport får endast återges i sin helhet, om inte utförande laboratorium i förväg skriftligen godkänt annat. Resultaten relaterar endast till det insända provet.

DJ009	Tryptofan	**0.317** g/100 g		EU 152/2009	*EUDAKG*
LP056	C 6:0 (Kapronsyra)	**<0.1** % av fettsyror	± 20%	Internal Method - GC-FID	*EUSELI*
LP056	C 8:0 (Kaprylsyra)	**<0.1** % av fettsyror	± 20%	Internal Method - GC-FID	*EUSELI*
LP056	C 10:0 (Kaprinsyra)	**1.2** % av fettsyror	± 20%	Internal Method - GC-FID	*EUSELI*
LP056	C 12:0 (Laurinsyra)	**48.1** % av fettsyror	± 10%	Internal Method - GC-FID	*EUSELI*
LP056	C 14:0 (Myristinsyra)	**6.8** % av fettsyror	± 10%	Internal Method - GC-FID	*EUSELI*
LP056	C 14:1 n-5 (Myristoleinsyra)	**<0.1** % av fettsyror	± 20%	Internal Method - GC-FID	*EUSELI*
LP056	C 15:0 (Pentadekansyra)	**0.2** % av fettsyror	± 20%	Internal Method - GC-FID	*EUSELI*
LP056	C 15:1 n-5	**<0.1** % av fettsyror	± 20%	Internal Method - GC-FID	*EUSELI*
LP056	C 16:0 (Palmitinsyra)	**13.9** % av fettsyror	± 10%	Internal Method - GC-FID	*EUSELI*
LP056	C 16:1 n-7 (Palmitoleinsyra)	**2.3** % av fettsyror	± 20%	Internal Method - GC-FID	*EUSELI*
LP056	C 17:0 (Margarinsyra)	**0.1** % av fettsyror	± 20%	Internal Method - GC-FID	*EUSELI*
LP056	C 17:1 n-7 (Heptadecensyra)	**<0.1** % av fettsyror	± 20%	Internal Method - GC-FID	*EUSELI*
LP056	C 18:0 (Stearinsyra)	**1.8** % av fettsyror	± 20%	Internal Method - GC-FID	*EUSELI*
LP056	C 18:1 (Oljesyra)	**13.2** % av fettsyror	± 10%	Internal Method - GC-FID	*EUSELI*
LP056	C 18:2 n-6 (Linolsyra)	**7.9** % av fettsyror	± 10%	Internal Method - GC-FID	*EUSELI*
LP056	C 18:3 n-3 (α-Linolensyra)	**1.5** % av fettsyror	± 20%	Internal Method - GC-FID	*EUSELI*
LP056	C 18:3 n-6 (γ-Linolensyra)	**<0.1** % av fettsyror	± 20%	Internal Method - GC-FID	*EUSELI*
LP056	C 18:4 n-3 (Oktadekatetraensyra)	**<0.1** % av fettsyror	± 20%	Internal Method - GC-FID	*EUSELI*
LP056	C 20:0 (Arachinsyra)	**<0.1** % av fettsyror	± 20%	Internal Method - GC-FID	*EUSELI*
LP056	C 20:1 n-9 (Gadoljesyra)	**<0.1** % av fettsyror	± 20%	Internal Method - GC-FID	*EUSELI*
LP056	C 20:2 n-6 (Eikosadiensyra)	**<0.1** % av fettsyror	± 20%	Internal Method - GC-FID	*EUSELI*
LP056	C 20:3 n-6	**<0.1** % av fettsyror	± 20%	Internal Method - GC-FID	*EUSELI*
LP056	C 20:3 n-3	**<0.1** % av fettsyror	± 20%	Internal Method - GC-FID	*EUSELI*
LP056	C 20:4 n-6 (Arakidonsyra)	**0.3** % av fettsyror	± 20%	Internal Method - GC-FID	*EUSELI*
LP056	C 20:4 n-3	**<0.1** % av fettsyror	± 20%	Internal Method - GC-FID	*EUSELI*
LP056	C 20:5 n-3 (EPA)	**0.4** % av fettsyror	± 20%	Internal Method - GC-FID	*EUSELI*
LP056	C 22:0 (Behensyra)	**<0.1** % av fettsyror	± 20%	Internal Method - GC-FID	*EUSELI*
LP056	C 22:1	**<0.1** % av fettsyror	± 20%	Internal Method - GC-FID	*EUSELI*
LP056	C 22:2 n-6 (Dokosadien syra)	**<0.1** % av fettsyror	± 20%	Internal Method - GC-FID	*EUSELI*
LP056	C 22:4 n-6	**<0.1** % av fettsyror	± 20%	Internal Method - GC-FID	*EUSELI*
LP056	C 22:5 n-6	**<0.1** % av fettsyror	± 20%	Internal Method - GC-FID	*EUSELI*
LP056	C 22:5 n-3 (Dokosapentaensyra)	**<0.1** % av fettsyror	± 20%	Internal Method - GC-FID	*EUSELI*
LP056	C 22:6 n-3 (DHA)	**<0.1** % av fettsyror	± 20%	Internal Method - GC-FID	*EUSELI*
LP056	C 24:0 (Lignoserinsyra)	**<0.1** % av fettsyror	± 20%	Internal Method - GC-FID	*EUSELI*
LP056	C 24:1 n-9 (Tetracosensyra)	**<0.1** % av fettsyror	± 20%	Internal Method - GC-FID	*EUSELI*
LP056	Summa mättade fettsyror	**72.1** % av fettsyror		Internal Method - GC-FID	*EUSELI*
LP056	Summa enkelomättade fettsyror	**15.6** % av fettsyror		Internal Method - GC-FID	*EUSELI*

Laboratoriet/laboratorierna är ackrediterade av respektive lands ackrediteringsorgan. Ej ackrediterade analyser är markerade med *

eurofins

Rapport utfärdad av
ackrediterat laboratorium

Report issued by
Accredited Laboratory

SWEDAC ACKREDITERING
Ackred. nr. 1977
Provning
ISO/IEC 17025

Eurofins Food & Feed Testing Sweden (Lidköping)
Box 887
Sjöhagsg. 3
SE-53119 Lidköping
www.eurofins.se

Kungliga Tekniska Högskolan
Mikael Hedenqvist
Fiber och Polymerteknologi, Teknikringen 58
100 44 STOCKHOLM

AR-18-LW-028396-02

EUSELI-00194631

Kundnummer: LW9903597

Analysrapport

Denna analysrapport ersätter tidigare version(er)
Vänligen makulera tidigare erhållna rapporter

Provnummer:	525-2018-05150064
Provmärkning:	Larver, svart soldatfluga, ca. 300g råprotein, aminosyraprofil, fettsyraprofil
Provet ankom:	2018-05-15
Analysrapport klar:	2018-06-01
Analyserna påbörjades:	2018-05-15

	Analys	Resultat	Enhet	Mäto.	Metod/ref	Lab
DI004	Alanin	**1.02**	g/100 g		SS-EN ISO 13903:2005	*EUDAKG*
DI004	Arginin	**0.930**	g/100 g		SS-EN ISO 13903:2005	*EUDAKG*
DI004	Asparginsyra	**1.71**	g/100 g		SS-EN ISO 13903:2005	*EUDAKG*
DI004	Glutaminsyra	**1.79**	g/100 g		SS-EN ISO 13903:2005	*EUDAKG*
DI004	Glycin	**0.997**	g/100 g		SS-EN ISO 13903:2005	*EUDAKG*
DI004	Histidin	**0.516**	g/100 g		SS-EN ISO 13903:2005	*EUDAKG*
DI004	* Hydroxiprolin	**<0.0500 (LOQ)**	g/100 g		SS-EN ISO 13903:2005	*EUDAKG*
DI004	Isoleucin	**0.770**	g/100 g		SS-EN ISO 13903:2005	*EUDAKG*
DI004	Leucin	**1.25**	g/100 g		SS-EN ISO 13903:2005	*EUDAKG*
DI004	Lysin	**1.13**	g/100 g		SS-EN ISO 13903:2005	*EUDAKG*
DI004	Ornitin	**<0.0500 (LOQ)**	g/100 g		SS-EN ISO 13903:2005	*EUDAKG*
DI004	Fenylalanin	**0.772**	g/100 g		SS-EN ISO 13903:2005	*EUDAKG*
DI004	Prolin	**0.920**	g/100 g		SS-EN ISO 13903:2005	*EUDAKG*
DI004	Serin	**0.728**	g/100 g		SS-EN ISO 13903:2005	*EUDAKG*
DI004	Treonin	**0.710**	g/100 g		SS-EN ISO 13903:2005	*EUDAKG*
DI004	Tyrosin	**1.16**	g/100 g		SS-EN ISO 13903:2005	*EUDAKG*
DI004	Valin	**1.06**	g/100 g		SS-EN ISO 13903:2005	*EUDAKG*
DJ011	Cystein +Cystine	**0.0980**	g/100 g		SS-EN ISO 13903:2005	*EUDAKG*
DJ011	Metionin	**0.346**	g/100 g		SS-EN ISO 13903:2005	*EUDAKG*
DJA70	* Summa aminosyror	**15.9**	g/100 g			*EUDAKG*
LP021	Råprotein enl. Kjeldahl (Nx6.25)	**18.1**	g/100 g	± 10%	NMKL 6:2003	*EUSELI*

Laboratoriet/laboratorierna är ackrediterade av respektive lands ackrediteringsorgan. Ej ackrediterade analyser är markerade med *

Förklaringar
* Ej ackrediterad analys

AR-003 v80
1.75 130516

Mäto: Mätosäkerhet

Mätosäkerheten, om inget annat anges, redovisas som utvidgad mätosäkerhet med täckningsfaktor 2. Undantag relaterat till analyser utförda utanför Sverige kan förekomma. Ytterligare upplysningar kan lämnas på begäran. Upplysning om mätosäkerhet och detektionsnivåer för mikrobiologiska analyser lämnas på begäran.

Denna rapport får endast återges i sin helhet, om inte utförande laboratorium i förväg skriftligen godkänt annat. Resultaten relaterar endast till det insända provet.

LP056	Summa fleromättade fettsyror	**10.1**	% av fettsyror	Internal Method - GC-FID	*EUSELI*
LP056	Totalsumma fettsyror	**97.8**	% av fettsyror	Internal Method - GC-FID	*EUSELI*
LP056	Oidentifierat	**2.2**	% av fettsyror	Internal Method - GC-FID	*EUSELI*
LP056	Summa av omega 6 fettsyror	**8.2**	% av fettsyror	Internal Method - GC-FID	*EUSELI*
LP056	Summa av omega 3 fettsyror	**1.9**	% av fettsyror	Internal Method - GC-FID	*EUSELI*
LP056	Kvot omega6/omega3 fettsyror	**4.31**		Internal Method - GC-FID	*EUSELI*

Rapportkommentar:
Trytofan tillagt enligt kunds önskemål.

Helena Byström, Rapportansvarig

Denna rapport är elektroniskt signerad.

Utförande Laboratorium

EUDAKG Eurofins Vitamin Testing Denmark, Vejen
EUSELI Eurofins Food & Feed Testing Sweden (Lidköping)

Laboratoriet/laboratorierna är ackrediterade av respektive lands ackrediteringsorgan. Ej ackrediterade analyser är markerade med *

Förklaringar
* Ej ackrediterad analys
Mäto: Mätosäkerhet

Mätosäkerheten, om inget annat anges, redovisas som utvidgad mätosäkerhet med täckningsfaktor 2. Undantag relaterat till analyser utförda utanför Sverige kan förekomma. Ytterligare upplysningar kan lämnas på begäran. Upplysning om mätosäkerhet och detektionsnivåer för mikrobiologiska analyser lämnas på begäran.

Denna rapport får endast återges i sin helhet, om inte utförande laboratorium i förväg skriftligen godkänt annat. Resultaten relaterar endast till det insända provet.
